# Spindle Checkpoint Regulators in Insulin Signaling

**DOI:** 10.3389/fcell.2018.00161

**Published:** 2018-11-29

**Authors:** Eunhee Choi, Hongtao Yu

**Affiliations:** Department of Pharmacology, Howard Hughes Medical Institute, University of Texas Southwestern Medical Center, Dallas, TX, United States

**Keywords:** spindle checkpoint, mitosis, insulin signaling, endocytosis, insulin receptor, evolutionary repurposing

## Abstract

The spindle checkpoint ensures accurate chromosome segregation during mitosis and guards against aneuploidy. Insulin signaling governs metabolic homeostasis and cell growth, and its dysregulation leads to metabolic disorders, such as diabetes. These critical pathways have been extensively investigated, but a link between the two has not been established until recently. Our recent study reveals a critical role of spindle checkpoint regulators in insulin signaling and metabolic homeostasis through regulating endocytosis of the insulin receptor (IR). These findings have linked spindle checkpoint proteins to metabolic regulation, expanding the connection between cell division and metabolism. Here, we briefly review the unexpected roles of spindle checkpoint regulators in vesicle trafficking and insulin signaling.

## Introduction

During cell division, each chromosome is replicated, and the replicated sister chromosomes are divided equally into two daughter cells. Microtubules attach to the kinetochore, a large protein assembly on centromeres, and generate pulling force toward opposing spindle poles. The spindle checkpoint monitors the kinetochore-microtubule attachment and tension across sister kinetochores (Foley and Kapoor, 2013; Jia et al., 2013; London and Biggins, 2014; Musacchio, 2015). Dysfunction of the spindle checkpoint causes chromosome missegregation and aneuploidy, resulting in developmental defects, cancer, and premature aging (Bharadwaj and Yu, 2004; Holland and Cleveland, 2009; Pfau and Amon, 2012; Funk et al., 2016).

The spindle checkpoint inhibits a multi-subunit ubiquitin ligase called the anaphase promoting complex/cyclosome (APC/C) in complex with its mitotic activator CDC20 and delays chromosome segregation (Peters, 2006; Yu, 2007; Luo and Yu, 2008; Izawa and Pines, 2015). The mitotic checkpoint complex (MCC), consisting of MAD2, BUBR1, BUB3, and CDC20, is a major inhibitor of APC/C^CDC20^ (Sudakin et al., 2001; Mapelli and Musacchio, 2007; Yu, 2007; Luo and Yu, 2008). Unattached kinetochores catalyze the formation of MCC, which diffuses away from the kinetochores to inhibit cellular APC/C^CDC20^.

**FIGURE 1:**
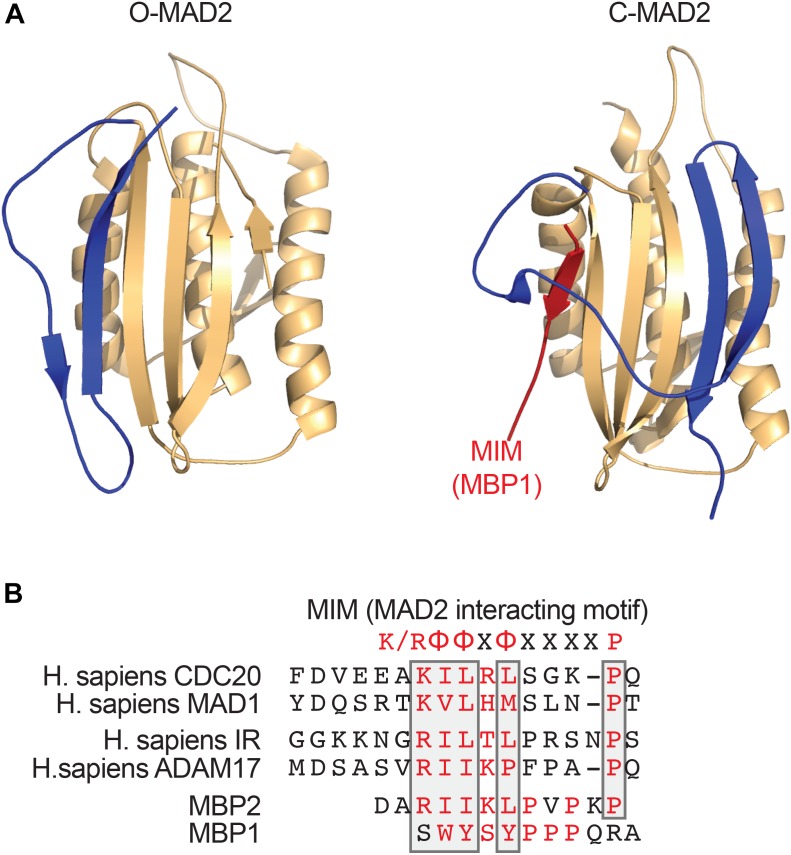
MAD2 binds short hydrophobic MAD2-interacting motif (MIM) motifs. **(A)** Ribbon diagrams of O-MAD2 (left, PDB ID 1DUJ) and C-MAD2 bound to the artificial MAD2-binding ligand MBP1 that was identified through phage display (right, PDB ID 1KLQ). The C-terminal region of MAD2 (colored in blue) undergoes a large conformational change to form the seat-belt structure that entraps its ligand. **(B)** Sequence alignment of the MIM from human CDC20, MAD1, insulin receptor (IR), ADAM17, and the MAD2-binding peptides (MBP1 and MBP2) that were identified through phase display. The key MAD2-binding residues are colored in red. The conserved residues in the MIM are boxed. The MIM consensus is shown on top. K/R denotes lysine or arginine; X, any residue; and Φ, a hydrophobic residue.

**FIGURE 2:**
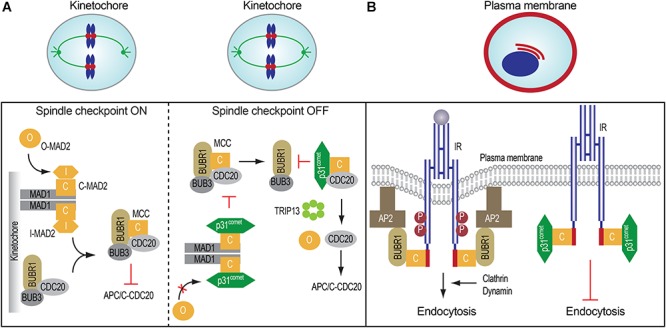
Spindle checkpoint proteins in mitosis and insulin signaling. **(A)** The roles and regulation of mitotic regulatory proteins in mitosis (left and middle). **(B)** MAD2, BUBR1, and p31^comet^ control insulin receptor endocytosis.

A critical step in the assembly of MCC is the conformational activation of MAD2. MAD2 has multiple conformations, including the inactive, open MAD2 (O-MAD2) and active, closed MAD2 (C-MAD2) (Figure 1A; Luo et al., 2002, 2004; Sironi et al., 2002; Mapelli and Musacchio, 2007; Luo and Yu, 2008). MAD2 binds to its activator MAD1 and its effector CDC20 through a short hydrophobic motif called the MAD2-interacting motif (MIM) with the consensus of (K/R)ΦΦXΦX_3-4_P (Φ, a hydrophobic residue; X, any residue) (Figure 1B). MAD2 holds the MIM through a seat-belt-like structure formed by its C-terminal region. Since the amino acid sequence of MIM is highly divergent, MAD2 can possibly interact with many proteins. The MAD1-C-MAD2 core complex at unattached kinetochores binds an additional copy of O-MAD2 and generates a conformation change of O-MAD2 to turn it into the intermediate MAD2 (I-MAD2) (Figure 2A). I-MAD2 entraps the MIM of CDC20 to form the stable CDC20-C-MAD2 complex. CDC20-C-MAD2 further binds with the constitutive BUBR1-BUB3 complex to form the intact MCC (Kulukian et al., 2009; Jia et al., 2013).

When all kinetochores are properly attached by microtubules, the MAD2-binding protein, p31^comet^, binds to MAD1-bound C-MAD2 and inhibits further MAD2 conformational activation, thus limiting MCC assembly (Figure 2A). In the MCC, the N-terminal region of BUBR1 directly contacts both CDC20 and C-MAD2 (Tipton et al., 2011; Chao et al., 2012; Tian et al., 2012). Because p31^comet^ and BUBR1 bind to a similar interface on C-MAD2, p31^comet^ competes with BUBR1 for C-MAD2 binding and weakens the contact between MAD2 and BUBR1 in the MCC. In addition, p31^comet^ recruits the AAA+ ATPase TRIP13, which induces the conformational change of C-MAD2 to O-MAD2 through the local unfolding of the C-terminal region of MAD2, thus releasing MAD2 from CDC20 and stimulating the disassembly of free MCC (Xia et al., 2004; Yang et al., 2007; Eytan et al., 2014; Wang et al., 2014; Ye et al., 2015, 2017; Brulotte et al., 2017; Alfieri et al., 2018). Finally, APC/C-mediated CDC20 ubiquitination and degradation trigger the disassembly of MCC already bound to APC/C^CDC20^ (Nilsson et al., 2008; Foster and Morgan, 2012; Uzunova et al., 2012; Yamaguchi et al., 2016). Collectively, these mechanisms promote APC/C^CDC20^ activation and anaphase onset. Therefore, dynamic assembly and disassembly of MCC are critical for timely chromosome segregation and genomic stability.

Although the components of the MCC are conserved from yeast to man, p31^comet^ is only found in metazoans. The yeast BUBR1 homolog, Mad3, lacks the C-terminal pseudokinase domain (Bolanos-Garcia and Blundell, 2011), which is dispensable for APC/C inhibition in human and mouse (Elowe et al., 2010; Suijkerbuijk et al., 2012). These findings suggest that p31^comet^ and the C-terminal domain of BUBR1 might have additional roles in multicellular organisms. In the mouse, MAD2 overexpression (Sotillo et al., 2007) or BUBR1 insufficiency (Baker et al., 2004) causes aneuploidy, but creates different physiological outcomes. Mice overexpressing MAD2 expectedly develop cancer, but mice with BUBR1 insufficiency exhibit premature aging. These results indicate that MAD2 and BUBR1 might control systemic tissue homeostasis beyond their functions in mitosis.

Our recent study has indeed uncovered a critical role of MAD2, BUBR1, and p31^comet^ in insulin signaling and metabolism (Choi et al., 2016). These mitotic regulators directly control insulin signaling and metabolic homeostasis through regulating endocytosis of the insulin receptor (IR). Here, we review our findings on the function and mechanism of the p31^comet^-MAD2-BUBR1 module in regulating IR endocytosis and insulin signaling, discuss the implications of these findings, and highlight key unanswered questions.

## Mitotic Regulatory Proteins in Glucose and Lipid Metabolism

The pancreas produces insulin to maintain metabolic homeostasis in vertebrates (Petersen and Shulman, 2018). Insulin binds to IR on the cell surface, disrupts the inactive IR dimer, and stabilizes the active dimer, in which the two cytoplasmic kinase domains undergo *trans*-autophosphorylation and activation (Gutmann et al., 2018; Scapin et al., 2018). The activated IR triggers two major signaling cascades: the phosphatidylinositol 3-kinase (PI3K)-AKT pathway and the mitogen-activated protein kinase (MAPK) pathway (White, 2003; Taniguchi et al., 2006; Boucher et al., 2014). The PI3K-AKT pathway controls glucose and lipid metabolism and the MAPK pathway governs cell growth. The activated IR can be internalized through clathrin-mediated endocytosis (CME), which attenuates insulin signaling at the plasma membrane (Goh and Sorkin, 2013).

Dysregulation of insulin signaling can cause insulin resistance syndromes, including type 2 diabetes. IR knockout (*Insr^-/-^*) mice exhibit mild growth retardation, glycogen storage defects, diabetic ketoacidosis, and neonatal lethality (Accili et al., 1996; Joshi et al., 1996). Liver-specific IR knockout (liver-*Insr^-/-^*) mice survive to adulthood and develop hepatic insulin resistance and dyslipidemia (Michael et al., 2000; Biddinger et al., 2008a,b). Thus, insulin signaling in the liver is critical for metabolic homeostasis.

Spindle checkpoint regulators are essential for embryonic viability in mice (Dobles et al., 2000; Kalitsis et al., 2000; Wang et al., 2004; Park et al., 2013). For example, loss of MAD2 causes early embryonic lethality, presumably due to severe chromosome missegregation and p53-dependent cell death during early development. Partial loss of the checkpoint increases aneuploidy, but produces variable phenotypes (Michel et al., 2001; Baker et al., 2004, 2009; Iwanaga et al., 2007; Jeganathan et al., 2007; Choi et al., 2012; Park et al., 2013). MAD2-overexpressing transgenic mice develop increased aneuploidy and spontaneous cancers, likely due to hyperactivation of the spindle checkpoint (Sotillo et al., 2007). Surprisingly, ablation of p31^comet^, a direct inhibitor of MAD2 function in the spindle checkpoint, causes hyperactivation of the spindle checkpoint, but does not produce the expected tumorigenesis phenotype. Instead, p31^comet^ knockout (*p31^-/-^*) mice exhibit mild growth retardation and neonatal lethality (Choi et al., 2016).

Multiple tissues of *p31^-/-^* mice examined, including heart, fat, and kidney, show no developmental abnormalities. Lung and respiratory muscle in *p31^-/-^* mice are normal, and there is evidence that the lungs have been inflated. Skeletal muscle is normal with intact sarcomeres, and the myofibers are not hypotrophic. *p31^-/-^* mice also exhibit normal liver development, including the formation of the hepatic cord and scattered, small hematopoietic foci. However, the glycogen level in hepatocytes, but not in the skeletal muscle, is significantly decreased in *p31^-/-^* mice. Glycogen stored in the liver is a crucial energy source (Girard and Pegorier, 1998). The insufficient energy to breathe and the inability to transition from placenta to nursing may have contributed to the neonatal lethality in *p31^-/-^* mice. The phenotypes of *p31^-/-^* mice are highly similar to those of *Insr^-/-^* mice and severe insulin resistance human diseases, such as Donohue syndrome (also known as leprechaunism) (Rogers, 1966).

Liver-specific p31^comet^ knockout (liver-*p31^-/-^*) mice survive to adulthood and show hyperinsulinemia and hyperglycemia that are less severe than those of liver-*Insr^-/-^* mice (Choi et al., 2016). Furthermore, despite having high serum insulin levels, liver-*p31^-/-^* mice show decreased levels of glycogen and triglyceride in the liver. Interestingly, their serum triglyceride levels are slightly increased, indicating that ablation of p31^comet^ in the liver can promote systemic changes in glucose and lipid metabolism. Like liver-*Insr^-/-^* mice, liver-*p31^-/-^* mice display glucose and insulin intolerance, albeit to lesser extent. These results suggest that p31^comet^ might promote insulin signaling.

p31^comet^ functions not only in the liver but also in other tissues, as *p31^-/-^* mice show neonatal lethality whereas liver-*p31^-/-^* mice are viable, a phenomenon also seen with whole-body or liver-specific IR ablation. The glycogen depletion in the liver of liver-*p31^-/-^* embryos is much less severe than that of the *p31^-/-^* embryos, allowing liver-*p31^-/-^* mice to survive. One obvious possibility is that insulin signaling in other tissues regulates hepatic glycogen levels. Incomplete genetic ablation of p31^comet^ by *Albumin-Cre* in embryos might be another contributing factor.

Disruption of BUBR1 causes early embryonic death accompanied by increased apoptosis (Wang et al., 2004). BUBR1 insufficiency (*Bub1b*^H/H^) mice expressing BUBR1 at approximately 10% the level of wild-type mice do not show discernible difference from wild-type littermates at birth, but develop aging-associated phenotypes, including cachexia, cataracts, and kyphosis (Baker et al., 2004). The fat deposits in *Bub1b*^H/H^ mice are greatly reduced, and they display muscle atrophy (Baker et al., 2004, 2008). Strikingly, BUBR1 insufficiency in mice improves glucose and insulin sensitivity without pancreatic β-cell degeneration (Baker et al., 2008; Choi et al., 2016).

In summary, ablation of the mitotic regulatory proteins triggers mitotic errors and increases aneuploidy. Because two aneuploidy mouse models (*p31^-^*^/^*^-^* and *Bub1b*^H/H^) have opposite metabolic phenotypes, in terms of glucose tolerance and insulin sensitivity, aneuploidy alone cannot underlie all metabolic phenotypes in these mice. Instead, these results suggest that mitotic regulators may directly control metabolic homeostasis *in vivo*.

## Mitotic Regulators in Insulin Signaling and Insulin Receptor Endocytosis

Clathrin-mediated endocytosis is an essential process in vesicle trafficking that transports various cargos from the cell surface to the inside of the cell (Goh and Sorkin, 2013; Traub and Bonifacino, 2013; Kaksonen and Roux, 2018). Over 50 soluble cytosolic proteins are involved in this process in a highly ordered manner. The assembly polypeptide 2 (AP2) complex is a key adaptor that links the clathrin lattice to both the cargo and lipid components of the plasma membrane. AP2 is a heterotetramer consisting of AP2A, AP2B1, AP2M1, and AP2S1 subunits. The entire AP2M1 and AP2S1 subunits, along with the N-terminal trunk domains of AP2A and AP2B1, make a large globular AP2 core. This core recognizes sorting signals of the cargo, such as acidic dileucine and YXXΦ (Y denotes Tyrosine; X, any residue; and Φ, a hydrophobic residue) endocytic motifs. The phosphoinositide, phosphatidylinositol 4,5-bisphosphate [PtdIns(4,5)P2], at the plasma membrane triggers a large conformational change of the AP2 core from the inactive “locked” form to the active “open” form, thus enabling cargo binding (Collins et al., 2002; Jackson et al., 2010). The C-terminal appendages of the AP2A and AP2B1 subunits extend from the core and bind to clathrin, other adaptors, and various accessory proteins, and promote clathrin vesicle formation.

The mechanism of IR endocytosis has been extensively studied for several decades. The kinase activity of IR is essential for its endocytosis (Grako et al., 1992; Carpentier et al., 1993). The NPEY motif in the juxtamembrane region and an acidic dileucine motif in the kinase domain of IR have been reported to promote IR endocytosis (Backer et al., 1990, 1991; Haft et al., 1994; Hamer et al., 1997). However, how the CME machinery can recognize the active IR and accelerate the clathrin pit formation is largely unknown.

Earlier studies implicated MAD2 and BUBR1 as IR- and AP2B1-interacting proteins, respectively, but the physiological functions of these interactions were not explored (O’Neill et al., 1997; Cayrol et al., 2002). The phenotypes of *p31^-^*^/^*^-^* mice suggested the possible involvement of p31^comet^ in insulin signaling and promoted us to re-examine the potential functions of these interactions. Our recent finding indicates that IR directly binds to MAD2 through a conserved MIM in the extreme C-terminal region (Figure 2B; Choi et al., 2016). IR-bound MAD2 adopts the active closed conformation (C-MAD2), similar to MAD1- or CDC20-bound C-MAD2. *In vitro* and in cells, the IR-bound C-MAD2 recruits AP2B1 through BUBR1, and promotes clathrin-mediated endocytosis of IR. As in inhibition of the spindle checkpoint signaling in mitosis, p31^comet^ blocks the association of MAD2-BUBR1-AP2B1 with IR, thereby inhibiting IR endocytosis. As revealed by total internal reflection fluorescence (TIRF) microscopy, p31^comet^, MAD2, and BUBR1 can indeed localize to the plasma membrane. The colocalization between IR and BUBR1 at the cell surface is increased by the chemical inhibition of dynamin, the GTPase required for clathrin-mediated endocytosis, with or without insulin treatment. Thus, the p31^comet^-MAD2-BUBR1 module regulates clathrin-mediated endocytosis of IR and insulin signaling in human cells.

Consistent with the *in vitro* findings, IR is prematurely internalized in liver from liver-*p31^-/-^* mice, resulting in insulin signaling defects and diabetic phenotypes (Choi et al., 2016). In contrast, IR autophosphorylation and activating AKT phosphorylation in response to insulin are more robust in *Bub1b*^H/H^ hepatocytes. Suppression of CME by depletion of AP2B1 or clathrin, or ablation of MAD2 or BUBR1 restores the level of IR on the plasma membrane and proper insulin signaling in *p31^-/-^* hepatocytes. Expression of MAD2-binding-defective IR rescues the insulin signaling defects and metabolic phenotypes in liver-*p31^-/-^* mice. Finally, liver-specific p31^comet^ and BUBR1 double knockout mice survive to adulthood and exhibit improved insulin sensitivity, similar to *Bub1b*^H/H^ mice.

Taken together, these findings establish a direct function of spindle checkpoint proteins in IR endocytosis and insulin signaling. This work provides a clear example of the evolutionary repurposing of a core cell division module for metabolic regulation. It further raises the interesting question why spindle checkpoint proteins are used to control insulin signaling.

The connection between mitotic regulators and vesicle trafficking is not limited to MAD2 and BUBR1. It has been reported that BUB1 can also bind to AP2B1 in a yeast-two hybrid assay (Cayrol et al., 2002). Recently, the *Drosophila* homolog of BUB1 has been shown to promote viral and pathogen entry into fly cells through mediating clathrin-mediated endocytosis (Yang et al., 2018). In that system, BUB1 physically interacts with the AP2 adaptor. Whether this function of BUB1 is conserved in mammals remains to be demonstrated. Unlike BUBR1, BUB1 is a functional kinase. It will be interesting to test if the BUB1 kinase activity is required for vesicle trafficking and, if so, BUB1 may be a viable target for limiting viral infections.

## Effect of Ploidy on Hepatic Metabolism

Wild-type hepatocytes are naturally polyploid, which can suppress liver tumorigenesis and enhance the functional capacity of the liver (Duncan et al., 2010, 2012; Zhang et al., 2018). While earlier studies had claimed that aneuploidy is common in normal rodent and human liver (Duncan et al., 2010, 2012), recent single cell whole-genome sequencing analysis has revealed that there is no widespread aneuploidy in wild-type mouse hepatocytes (Knouse et al., 2014; Choi et al., 2016). Disruption of the spindle checkpoint is expected to generate aneuploidy. Several complementary lines of evidence argue against the change in ploidy as the determining factor of the metabolic defects in liver-*p31^-/-^* mice (Choi et al., 2016). First, the polyploidy status is not altered in liver-*p31^-/-^* hepatocytes. Second, single-cell sequencing analysis reveals that about 5% of liver-*p31^-/-^* hepatocytes and 20% of *Bub1b*^H/H^ hepatocytes are aneuploid. Thus, although ablation of p31^comet^ in mouse embryonic fibroblasts (MEFs) causes high incidences of aneuploidy similar to those previously reported for MEFs harboring a hypomorphic allele of CDC20 or BUBR1 (Baker et al., 2004; Malureanu et al., 2010), the aneuploidy incidence of *p31^-^*^/^*^-^* hepatocytes *in vivo* is surprisingly low. Importantly, the *Bub1b*^H/H^ mice harboring higher incidence of aneuploidy in hepatocytes show insulin sensitivity, as opposed to insulin resistance seen in liver-*p31^-/-^* mice. Third, re-expression of p31^comet^ in the adult liver rescues the metabolic phenotypes and insulin signaling defects of liver-*p31^-/-^* mice, without altering the low-level aneuploidy in hepatocytes. Similarly, expression of the MAD2-binding-deficient mutant of IR, but not wild-type IR, rescues the metabolic phenotypes of liver-*p31^-/-^* mice. These genetic suppression experiments provide the strongest evidence that aneuploidy is not the sole factor driving the metabolic phenotypes.

Collectively, these data strongly support the specific functions of the mitotic regulators in insulin signaling and metabolism. *Bub1b*^H/H^ mice undergo premature aging. Given the prominent roles of the insulin pathway in aging, it is conceivable that the hyperactive insulin pathway in *Bub1b*^H/H^ contributes to their premature aging phenotypes. This possibility needs to be further investigated in future studies.

## Summary and Outlook

The spindle checkpoint is critical for mitotic fidelity in dividing cells. Insulin signaling coordinates both metabolic homeostasis and cell proliferation. The p31^comet^-MAD2-BUBR1 module of crucial spindle checkpoint proteins plays an important role in insulin signaling and systemic homeostasis by ensuring timely IR endocytosis (Figure 2B). These unexpected findings raise many interesting questions: (1) How do this mitotic module and the known mechanisms of IR endocytosis cooperate to regulate IR endocytosis? (2) How does insulin stimulation suppress p31^comet^-mediated inhibition of BUBR1-AP2 association with IR? Can insulin signaling control this mitotic module? (3) Are the mitotic and metabolic functions of the spindle checkpoint regulators linked? Can extracellular hormones regulate chromosome segregation through IR? (4) What is the physiological consequence of disruption of the IR-MAD2 interaction? Can it promote tumorigenesis or suppress diabetic phenotypes? Future studies aimed at answering these questions will greatly advance our understanding of the physiological functions of the unexpected connection between checkpoint proteins and insulin signaling.

MAD2 interacts with IR through the MIM (Choi et al., 2016). The mitotic p31^comet^-MAD2-BUBR1 module likely only regulates cell-surface receptors that contain the MIM. The insulin-like growth factor 1 receptor (IGF1R) and IR share over 80% homology in their intracellular domains. However, IGF1R does not contain the MIM, and is unlikely to be regulated by the p31^comet^-MAD2-BUBR1 module. On the other hand, other unrelated receptors that contain the MIM may be regulated through similar mechanisms. For example, ADAM17/TACE, which is a metalloprotease with crucial functions in cancer biology, has a functional MIM and binds directly to MAD2 (Nelson et al., 1999; Murphy, 2008; Choi et al., 2016). It will be interesting to examine the potential regulation of ADAM17 by MAD2. Future studies are also required to systematically discover new MAD2-binding receptors and to elucidate the physiological functions of these binding events. Research in this direction may reveal new regulatory mechanisms of cell surface receptors, expand the non-cell-cycle functions of mitotic regulators, and uncover novel therapeutic targets for treating human diseases, such as cancer and diabetes.

## Author Contributions

EC wrote the initial draft of the review. HY made a substantial edits and finalized the article.

## Conflict of Interest Statement

The authors declare that the research was conducted in the absence of any commercial or financial relationships that could be construed as a potential conflict of interest.
